# Interventricular Septal Hematoma and Coronary-Ventricular Fistula: A Complication of Retrograde Chronic Total Occlusion Intervention

**DOI:** 10.1155/2016/8750603

**Published:** 2016-09-07

**Authors:** Abdul-rahman R. Abdel-karim, Minh Vo, Michael L. Main, J. Aaron Grantham

**Affiliations:** ^1^Saint Luke's Mid America Heart Institute, University of Missouri Kansas City, Kansas City, MO, USA; ^2^University of Manitoba, St. Boniface Hospital, Winnipeg, MB, Canada

## Abstract

Interventricular septal hematoma is a rare complication of retrograde chronic total occlusion (CTO) percutaneous coronary interventions (PCI) with a typically benign course. Here we report two cases of interventricular septal hematoma and coronary-cameral fistula development after right coronary artery (RCA) CTO-PCI using a retrograde approach. Both were complicated by development of ST-segment elevation and chest pain. One case was managed actively and the other conservatively, both with a favorable outcome.

## 1. Introduction

Interventricular septal hematoma is a rare complication of retrograde chronic total occlusion (CTO) percutaneous coronary interventions (PCI) with a typically benign course [1]. Here we report two cases of interventricular septal hematoma and coronary-cameral fistula development after right coronary artery (RCA) CTO-PCI using a retrograde approach. Both cases were complicated by the development of ST-segment elevation and chest pain. One case was managed actively with exclusion of the hematoma and the other conservatively, both with a favorable outcome. These cases with clues from seven other case reports have led us to a suggested observation and treatment algorithm for this unique procedural complication.

## 2. Case 1

**Figure 1:**
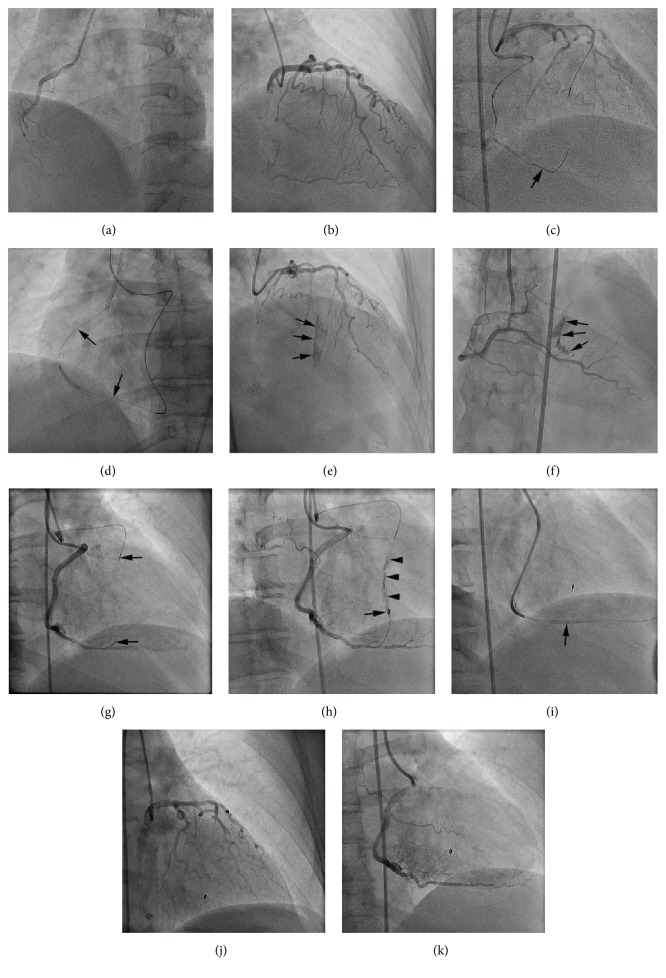
(a) Right coronary artery (RCA) chronic total occlusion (CTO). (b) Left anterior descending artery (LAD) supplying Werner's grade 1 septal collaterals to RCA CTO. (c) Fielder FC (arrow) advanced from LAD through second septal perforator into distal RCA. (d) After successful retrograde wire crossing, externalization was achieved with guidewire (arrow). (e and f) Angiogram of LAD and RCA, respectively, reveals Ellis Type III septal collateral perforation (arrows) into the right ventricle. (g) Negative suction applied to Corsairs (arrow heads) that were advanced from LAD and RCA into the perforated septal collateral. (h) Detachable coil delivered (arrow) but persistent of perforation seen (arrow head). (i) Covered stent (arrow) delivered across perforated septal vessel. (j and k) Final angiogram of LAD and RCA, respectively, confirmed resolution of septal perforation.

A 46-year-old female with Canadian Cardiovascular Society (CCS) class IV angina symptom despite maximal medical therapy was referred for coronary angiogram and was found to have right coronary artery (RCA) chronic total occlusion (CTO) (Figure 1(a)).

RCA CTO percutaneous intervention was performed using right radial and right femoral artery dual catheter approach. The RCA was engaged with 6 French Judkins right guide catheter; the right femoral artery was cannulated with a 6 French sheath and the left main was engaged with a 6 French XB 3.5 guide catheter. With standard antegrade wire escalation technique, the CTO could not be crossed [2]. The treatment strategy was changed to the retrograde approach utilizing septal collaterals from the left anterior descending artery (LAD) (Figure 1(b)). A fielder FC guidewire (Asahi Intecc, Nagoya, Japan) was advanced through the second septal perforator and then into the distal RCA (Figure 1(c)). This was followed by advancement of a Corsair catheter (Asahi Intecc, Nagoya, Japan) into the distal RCA. A Pilot 200 (Abbott Vascular, Abbott Park, Illinois) was advanced through the Corsair and crossed the CTO segment into the antegrade guide catheter followed by Corsair advancement into the guide. An R350 guidewire (Vascular Solutions, Minneapolis, Minnesota) was used for externalization (Figure 1(d)) and three drug-eluting stents were delivered.

After removal of retrograde Corsair and guidewire, angiogram of the LAD and RCA revealed Ellis Type III cavity spilling perforation of the second septal branch into the right ventricle (RV) (Figures 1(e) and 1(f)). The patient was asymptomatic and hemodynamically stable and therefore transferred to a monitored bed. Subsequently, she complained of intermittent chest pain throughout the night and had sinus tachycardia with normal blood pressure.

The next morning, she was taken back to the cardiac catheterization lab and repeat coronary angiography appeared unchanged. Given the patient's symptom and tachycardia, the decision to proceed with exclusion of the septal artery to RV perforation was made. Bilateral femoral access was obtained and similar guide catheters were used to engage the left and right coronary systems. The perforated collateral septal branch was accessed with workhorse type coronary guidewires from the LAD and RCA. Corsairs were then advanced into the proximal segment of the collateral vessel and negative suction was applied for approximately ten minutes (Figure 1(g)). This maneuver was successful in resolving the perforation from the LAD but not from the RCA side. Therefore, a Renegade Microcatheter (Boston Scientific, Natick, Massachusetts) was advanced from the RCA into the perforated septal branch and a 2 × 3 × 23 mm complex helical Interlock detachable coil (Boston Scientific) was delivered but was ineffective (Figure 1(h)). An attempt to deliver another coil failed due to poor microcatheter support and, therefore, a 2.8 × 19 mm Graftmaster covered stent (Abbott Vascular) was deployed across the perforated collateral vessel in the posterior descending artery of the RCA (Figure 1(i)). Final angiograms of the left and right coronary system confirmed complete resolution of the perforation (Figures 1(j) and 1(k)). The patient's chest pain and tachycardia resolved after the procedure and she was subsequently discharged home.

## 3. Case 2

A 66-year-old male with known coronary artery disease and a chronically occluded mid right coronary stent presented with non-ST-segment elevation myocardial infarction. The culprit lesion was the RCA proximal to the remotely deployed and chronically occluded bare metal stent resulting in right ventricular ischemia. An attempt to recanalize the RCA was unsuccessful. He was treated medically but had persistent Canadian Cardiovascular Society (CCS) class III angina and was referred for RCA CTO-PCI.

The antegrade approach was unsuccessful because the wire repeatedly tracked beside the stent; therefore, the retrograde approach was used. The in-stent lesion was successfully crossed with reverse cart technique in the proximal RCA [8]. The RCA was then stented with 2.5 × 38 mm, 3.0 × 38 mm, 3.5 × 38 mm, and 3.5 × 8 mm drug-eluting stents from distal to proximal, respectively. TIMI 3 flow was achieved after procedure.

**Figure 2:**
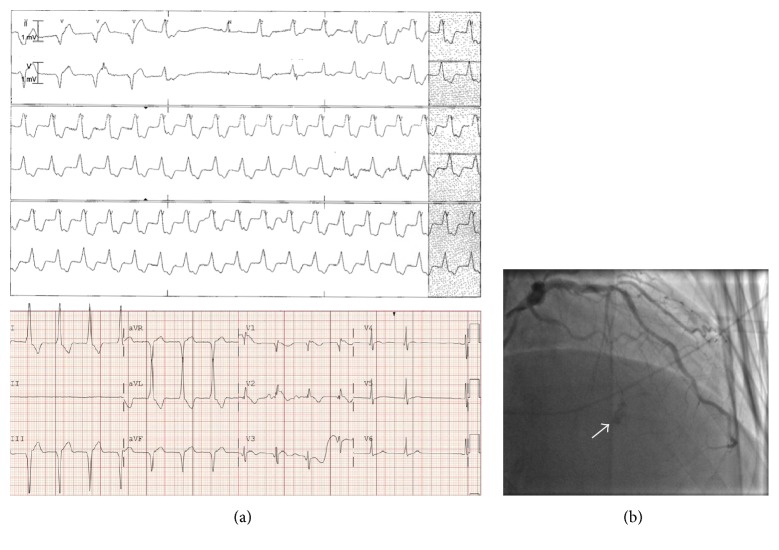
(a) A cardiac telemetry monitor strip and an electrocardiogram showing nonsustained ventricular tachycardia and anterior ST-segment elevation after RCA CTO-PCI using the retrograde approach, which was complicated by perforation of first septal artery branch (arrow, (b)).

On the night after his PCI, he developed severe chest pain, anterior ST-segment elevation, and nonsustained ventricular tachycardia (Figure 2(a)). Repeat angiography showed patent RCA stents and a perforation with large septal hematoma formation in the first septal artery branch territory (Figure 2(b)). He was treated conservatively with beta blockers and analgesics.

**Figure 3:**
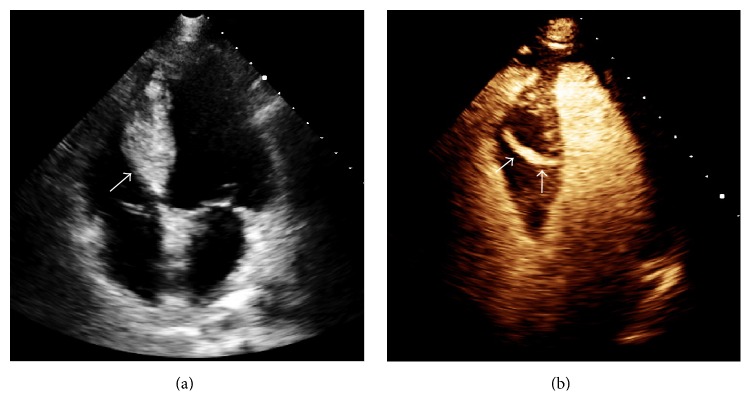
Interventricular septal hematoma (5.3 × 2.6 cm) (arrow, (a)), with coronary-ventricular fistula (5 mm in diameter) (arrows, (b)). (Videos 1 and 2 show baseline echocardiographic findings and are included in Supplementary Material available online at http://dx.doi.org/10.1155/2016/8750603.)

The following day, a contrast enhanced transthoracic echocardiogram confirmed a new large interventricular hematoma. The end systolic dimensions were 5.3 × 2.6 cm (Figure 3(a)). There was a 5 mm diameter fistulous tract from the left ventricle that appeared to extend to within a few millimeters of the right ventricular cavity (Figure 3(b)). Serial echocardiography revealed no appreciable change in the hematoma and fistula. There was no further arrhythmia and right heart catheterization with oximetry showed a Qp/Qs of 1.1 : 1. The patient was discharged on hospital day four.

**Figure 4:**
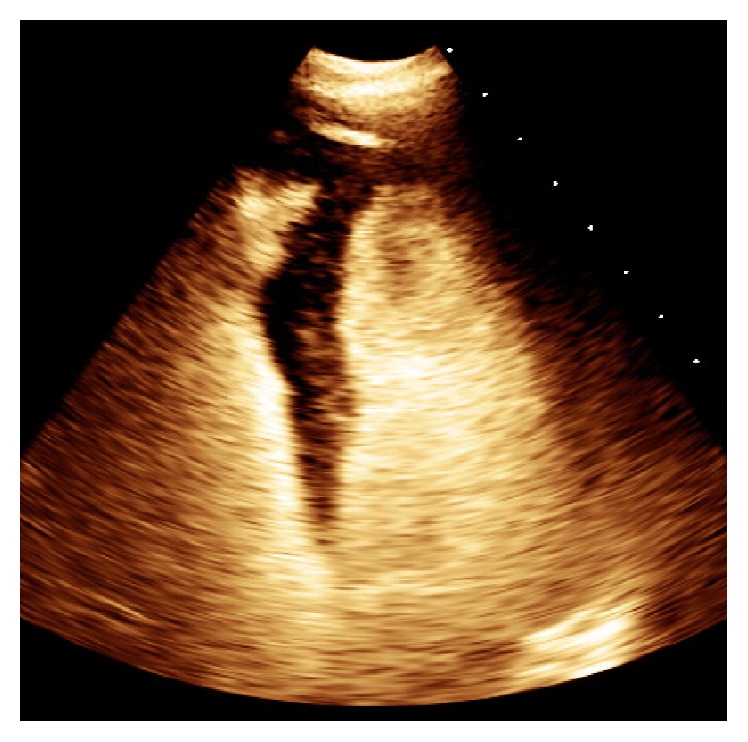
Complete resolution of interventricular septal hematoma at 3 months after PCI. (Video 3 shows follow-up echocardiographic findings and is included as supplementary material online.)

Ten days after PCI he was clinically stable and angina-free. An echocardiogram revealed a smaller intramyocardial septal hematoma (3.6 × 1.3 cm) with a fistulous tract in the septum that entered the apical right ventricle. At 3 months a subsequent echocardiogram showed resolution of the interventricular septal hematoma (Figure 4) and the patient remained symptom-free.

## 4. Discussion

CTO-PCI frequency is increasing [9]. In a recent meta-analysis including 26 studies, use of the retrograde approach was associated with a technical success rate of 74.5% (overall CTO-PCI procedural success rate was 83.3%). Typical procedural major adverse cardiovascular events (MACE) including mortality (0.7%), urgent CABG (0.7%), and stroke (0.5%) were described. CTO specific complications such as collateral perforation occurred in 6.9% of cases with tamponade occurring in 1.4% [10].

Septal collateral perforation and hematoma formation is a rare complication after retrograde CTO-PCI [1] and believed to have a typically benign course especially among asymptomatic cases. Here we present two cases where clinical deterioration led to further evaluation and subsequent intervention in one but not the other. In both cases there were no adverse sequelae but aggressive management led to additional treatment with coils and a covered stent while conservative management led to prolonged inpatient observation and additional imaging. Other individual case reports of septal hematoma after retrograde CTO-PCI exist (Table 1). In most of the reported cases, cardiac imaging such as contrast and Doppler two-dimensional echocardiography, computed tomography, and cardiac magnetic resonance imaging played a crucial role in early detection and monitoring of these complications.

**Figure 5:**
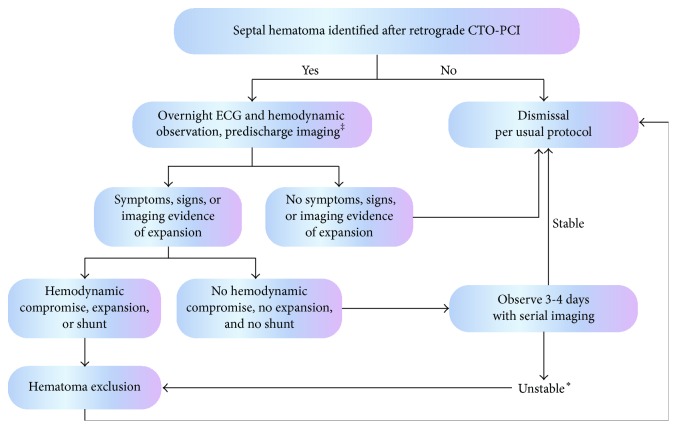
Suggested algorithm for observation and management of septal hematoma after retrograde CTO-PCI. ^‡^Imaging can be performed with catheterization, contrast echocardiography (our preferred method), or perhaps cardiac magnetic resonance imaging. ^*∗*^Unstable includes hemodynamic compromise, continued symptoms, development of large shunt, persistent or recurrent life threatening arrhythmia, effusion, or tamponade.

While the management of these cases remains controversial and undefined by large experience some guidance is needed as the frequency of CTO-PCI and the retrograde approach increases. Taking our observations together with previous reports we suggest the following when retrograde CTO-PCI results in septal hematoma formation (Figure 5). Inpatient telemetry, hemodynamic, and Creatine Kinase (CK) and CK-MB levels monitoring of these patients for symptoms and signs of hematoma expansion is prudent.

Patients who remain asymptomatic can be managed conservatively and dismissed 24–48 hours after CTO-CPI. Among those with symptoms of hematoma expansion, those without hemodynamic compromise can also be monitored with serial echocardiography and dismissed after 3-4 days of additional observation in the absence of additional evidence of hematoma expansion or significant shunt formation. We recommend more aggressive management with hematoma exclusion for those with hemodynamic compromise, evidence of additional hematoma expansion, pericardial effusion, tamponade, or significant shunt development (Qp/Qs > 1.5 : 1). Early dismissal after definitive management is not unreasonable and may be useful in avoiding prolonged inpatient observation, but the long term outcome of this approach is unknown. After successful CTO-PCI, exclusion of septal hematoma can be accomplished by local occlusion of the contributing septal perforator or occlusion of the origin of the septal with a covered stent. Both the donor and recipient vessel side of the collateral must be occluded to prevent flow into the hematoma. After unsuccessful CTO-PCI only the donor limb of the collateral needs to be occluded. Local occlusion of either limb of the septal can be accomplished with aspiration (as in Case 1) through a microcatheter or coiling with a bailout strategy of covered stenting. We preferentially use aspiration and coils in an effort to avoid the long term risk of covered stent restenosis or thrombosis which is as high as 30% in some series [11]. Additionally, coil occlusion of the septal hematoma space is possible. Caution should be used with microsphere, thrombus, gelfoam, or thrombin injection thrombosis of the hematoma space as many of these communicate with a ventricular cavity and extrusion of these materials into the cavity is a potentially catastrophic possibility.

## 5. Conclusion

Septal hematoma formation is an unusual but potentially dangerous complication of the increasingly used technique of retrograde CTO-PCI. The case reports to date provide some clues as to the best management of this event. We propose an observation and treatment algorithm and recommend serial imaging with contrast echocardiography to assist in the decision-making during these rare but important events.

## Supplementary Material

RCA CTO PCI was complicated by large interventricular septal hematoma “5.3 × 2.6 cm” at end systole (video 1), with coronary-ventricular fistula (5 mm in diameter) (Video 2). At 3 months post PCI a subsequent echocardiogram showed resolution of the hematoma (Video 3).

## Figures and Tables

**Table 1:** Other individual case reports of septal hematoma after retrograde CTO-PCI.

Author	Publication year	CTO location and approach	Signs and symptoms	Complications	Intervention	Imaging modality used
Fairley et al. [1]	2010	LAD, retrograde	Asymptomatic, ventricular bigeminy	Ventricular Septal Defect (VSD)	Spontaneous resolution at 5 weeks	Echocardiography

Murthy et al. [3]	2014	LAD in-stent restenosis, antegrade	Contrast stain during catheterization, asymptomatic	Coronary-cameral fistula	Covered stent	Coronary angiography and Intravascular Ultrasound (IVUS)

Lin et al. [4]	2006	LAD, retrograde	Fever and chest pain	Myocardial infarction and septal hematoma	Spontaneous resolution at 1 month	Echocardiography and computed tomography

Hashidomi and Saito [5]	2011	LAD in-stent restenosis, retrograde	Contrast stain, immediate hypotension and tachycardia	Cardiac tamponade	Pericardiocentesis and coil embolization	Echocardiography

Higuchi et al. [6]	2015	LAD, antegrade	Contrast stain, cardiogenic shock	Large expanding subepicardial hematoma, tamponade, and death	Attempted pericardial drainage	Echocardiography

Araki et al. [7]	2016	RCA, retrograde	Contrast stain, asymptomatic	None	Spontaneous resolution at 6 weeks	Echocardiography and cardiac magnetic resonance imaging

## Abbreviations

CTO: Chronic total occlusion
PCI: Percutaneous coronary interventions
RCA: Right coronary artery
LAD: Left anterior descending
CCS: Canadian Cardiovascular Society
RV: Right ventricle
Qp/Qs: Pulmonary flow/systemic flow
MACE: Major adverse cardiovascular events
CABG: Coronary artery bypass graft
VSD: Ventricular Septal Defect
NSVT: Nonsustained ventricular tachycardia
EKG: Electrocardiogram.
